# Additional annotation enhances potential for biologically-relevant analysis of the Illumina Infinium HumanMethylation450 BeadChip array

**DOI:** 10.1186/1756-8935-6-4

**Published:** 2013-03-03

**Authors:** E Magda Price, Allison M Cotton, Lucia L Lam, Pau Farré, Eldon Emberly, Carolyn J Brown, Wendy P Robinson, Michael S Kobor

**Affiliations:** 1 Department of Obstetrics and Gynaecology, University of British Columbia, 2H30-4490 Oak Street, Vancouver, BC, V6H 3N1, Canada; 2 The Child & Family Research Institute, 950 West 28th Avenue, Vancouver, BC, V5Z 4H4, Canada; 3 Department of Medical Genetics, University of British Columbia, 2329 West Mall, Vancouver, BC, V6T 1Z3, Canada; 4 Centre for Molecular Medicine and Therapeutics, 950 West 28th Avenue, Vancouver, BC, V5Z 4H4, Canada; 5 Department of Physics, Simon Fraser University, 8888 University Drive, Burnaby, BC, V5A 1S6, Canada

**Keywords:** Infinium HumanMethylation450 BeadChip array, DNA methylation, non-specific probes, Polymorphic probes, CpG islands, Annotation, CpG enrichment, Tissue-specific DNA methylation, Repetitive elements, 450 k

## Abstract

**Background:**

Measurement of genome-wide DNA methylation (DNAm) has become an important avenue for investigating potential physiologically-relevant epigenetic changes. Illumina Infinium (Illumina, San Diego, CA, USA) is a commercially available microarray suite used to measure DNAm at many sites throughout the genome. However, it has been suggested that a subset of array probes may give misleading results due to issues related to probe design. To facilitate biologically significant data interpretation, we set out to enhance probe annotation of the newest Infinium array, the HumanMethylation450 BeadChip (450 k), with >485,000 probes covering 99% of Reference Sequence (RefSeq) genes (National Center for Biotechnology Information (NCBI), Bethesda, MD, USA). Annotation that was added or expanded on includes: 1) documented SNPs in the probe target, 2) probe binding specificity, 3) CpG classification of target sites and 4) gene feature classification of target sites.

**Results:**

Probes with documented SNPs at the target CpG (4.3% of probes) were associated with increased within-tissue variation in DNAm. An example of a probe with a SNP at the target CpG demonstrated how sample genotype can confound the measurement of DNAm. Additionally, 8.6% of probes mapped to multiple locations *in silico*. Measurements from these non-specific probes likely represent a combination of DNAm from multiple genomic sites. The expanded biological annotation demonstrated that based on DNAm, grouping probes by an alternative high-density and intermediate-density CpG island classification provided a distinctive pattern of DNAm. Finally, variable enrichment for differentially methylated probes was noted across CpG classes and gene feature groups, dependant on the tissues that were compared.

**Conclusion:**

DNAm arrays offer a high-throughput approach for which careful consideration of probe content should be utilized to better understand the biological processes affected. Probes containing SNPs and non-specific probes may affect the assessment of DNAm using the 450 k array. Additionally, probe classification by CpG enrichment classes and to a lesser extent gene feature groups resulted in distinct patterns of DNAm. Thus, we recommend that compromised probes be removed from analyses and that the genomic context of DNAm is considered in studies deciphering the biological meaning of Illumina 450 k array data.

## Background

Measuring epigenetic marks has become an attractive approach for connecting phenotype, genetics and environment in many fields of medicine [1-3]. DNA methylation (DNAm), the addition of a methyl group primarily to cytosines in the context of CpG dinucleotides, is one such highly studied epigenetic mark. Epigenome-wide association studies (EWAS) of DNAm have been proposed as a complement to genome-wide association studies (GWAS) for elucidating loci correlated with complex disease [4]. Although these large-scale association studies provide a great amount of information, there are currently limits to our ability to interpret this data [5] given the variability of DNAm across individuals, ethnicities, sex, age, tissue type and environment [6,7]. To improve the analysis potential of a popular tool for large-scale measurement of DNAm, the Infinium HumanMethylation450 BeadChip (450 k) (Illumina, San Diego, CA, USA), we have annotated technically unreliable probes and enhanced the biological annotation of this DNAm microarray.

The 450 k array combines two technically distinct assays in one platform: the Infinium I assay (type I probes) and Infinium II assay (type II probes) (see methods section for details). The design and specifications of the 450 k array have been discussed in other publications [8-10], and extensive probe annotation is available from Illumina to aid users in data interpretation. This annotation includes, for example, probe location within genes (annotated by University of California, Santa Cruz (UCSC) Genome Browser (http://genome.ucsc.edu; UCSC Genome Bioinformatics, Santa Cruz, CA, USA), CpG islands and shores, and regulatory features. However, recently technical limitations of the Infinium platform have been described [11,12]. In 2011, an evaluation of an earlier version of the array, the Infinium HumanMethylation27k BeadChip (27 k) that used exclusively the Infinium I assay, identified two groups of probes as possibly compromised by their design [10]. The first group, accounting for about 6 to 10% of the 27 k array, was non-specific probes, that is, probes that hybridized to multiple genomic locations *in silico*. The level of DNAm at non-specific probes likely reflects a combination of DNAm at the various locations to which these probes hybridize. The second group of unreliable probes was those with a polymorphic target (0.24% of 27 k probes). Since the Infinium DNAm platform uses quantitative genotyping of C/T SNPs introduced following bisulfite conversion to determine the level of DNAm, probes with polymorphisms at the target C or G have the potential of assessing a difference in genotype rather than a true difference in DNAm. A corresponding increase in the number of both non-specific probes and polymorphic probes is expected given the similar technology of the 450 k array [12].

CpG dinucleotides are not randomly distributed throughout the genome, most have spontaneously deaminated with the exception of some CpG-enriched regions known as ‘CpG islands’ [13]. About 70% of gene promoters are associated with CpG islands [14] and traditionally gene transcription has been thought to be repressed by the presence of promoter CpG island DNAm [15,16]. There are different approaches for classifying CpG enrichment, for example, UCSC defines CpG islands based on CG content >50%, Observed/Expected (Obs/Exp) CpG ratio >0.6 and length >200 bps [17]. An alternative classification of CpG islands providing more enrichment discrimination is high-density CpG islands (HCs, CG content >55%, Obs/Exp CpG ratio >0.75 and length >500 bps), intermediate-density CpG islands (ICs, CG content >50%, Obs/Exp CpG ratio >0.48 and length >200 bps) and non-islands (LCs or low-density CpG regions, non-HC/IC regions) [16,18]. However, the most biologically meaningful definition of CpG enrichment remains to be determined.

In the past, many DNAm studies focused on promoters and CpG islands however, recently attention has also turned towards the study of DNAm patterns in the regions surrounding islands, known as shores. CpG island shores have been observed to be variably methylated between unrelated individuals, in cancer and in iPS cell lines [19-21]. The level of DNAm in shores may in fact be more highly correlated with gene expression than that of CpG islands [22]. Furthermore, tissue-specific gene expression has been associated with tissue differences in DNAm at shores [19], perhaps as a consequence of transcription machinery binding to nearby promoter CpG islands. Others have shown that DNAm outside of CpG islands and shores may also be associated with gene expression. For example, one study identified lower levels of gene body DNAm associated with the lowest and highest levels of gene expression, whereas higher levels of gene body DNAm were associated with intermediate levels of gene expression [23].

While the 450 k array offers the opportunity to examine DNAm at individual CpGs across CpG island and non-island regions, the inclusion of this diverse range of sites requires a more complex and detailed annotation of the array. To enhance the utility of the 450 k array, we increased probe annotation in four areas: 1) documented SNPs in the target CpG, 2) probe binding specificity, 3) CpG classification of target sites and 4) gene feature classification of target sites. We tested the expanded annotation in a set of control samples of interest to our investigations: adult blood (n = 4), child buccal (n = 4) and placental chorionic villi (n = 4), and followed up some analyses in a larger, publically available blood dataset (n = 261). In particular, we evaluated DNAm patterns relative to functional aspects of probe location, while considering the effects of technically biased probes. Based on our analyses, we recommend that users analyze 450 k data with the following factors in mind: 1) DNAm measured at probes with SNPs in the target site may be compromised by sample genotype, 2) DNAm measured at non-specific probes may not only represent the intended site of hybridization and 3) DNAm varies across CpG enrichment classes as well as gene features.

## Results and discussion

### Polymorphic CpGs may affect the assessment of DNAm

Infinium assays are based on quantifying bisulfite-introduced C/T SNPs, thus the actual DNA sequence at the target CpG is at risk of compromising the assessment of DNAm. The end of each 450 k probe targets a CpG of interest and although the alignment of type I and type II probes with CpGs differs by one base pair (Additional file 1), end nucleotide match is essential for extension of both probe types. A SNP leading to a sequence change at a target CpG might result in a false DNAm signal due to hybridization of the wrong probe (possible for type I probes) or no/minimal extension at the target site (possible for both probe types). Illumina included annotation of SNPs located within 10 bps of the target CpG (SNP <10 bp, n = 36,535 probes) and those located within the remainder of the probe (SNP >10 bp, n = 59,892 probes). We have added annotation of probes that query CpGs with documented polymorphisms specifically at the C and/or G position (target CpG SNPs).

**Figure 1:**
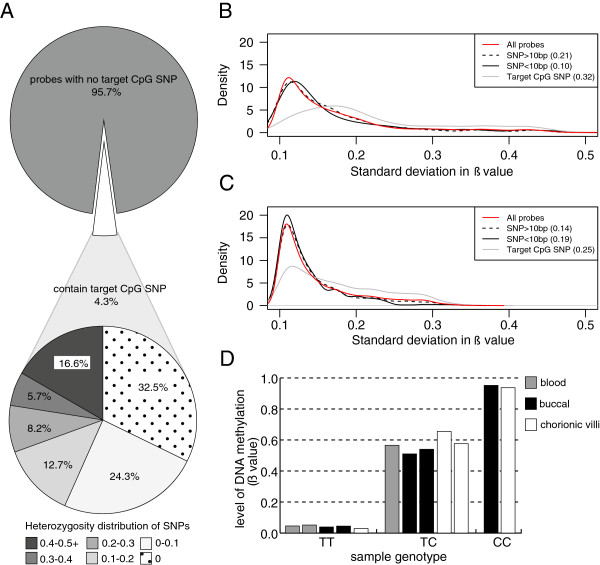
**Probes targeting polymorphic CpGs may affect the assessment of DNAm.** (**A**) A documented SNP was identified at the target C or G position of 4.3% of 450 k probes (target CpG SNP). Of these SNPs, 43.2% had a heterozygosity of >0.1 and due to their frequency in the population are more likely to affect measurement of DNAm. (**B**) Using blood samples (n = 4) as example, the SD in ß value between individuals was calculated for all probes. Probes with small SD in ß (<0.10) were removed from the analysis. The distribution of SD in ß value was plotted for all probes, and for the subsets of probes annotated with a target CpG SNP, a SNP within 10 bps of the target but without a target CpG SNP (SNP <10 bp) and a SNP within the remainder of the probe (SNP >10 bp). Numbers in brackets indicate Kolmogorov-Smirnov (KS) statistics in comparison to the distribution of all probes. (**C**) Using a selection of 261 adult blood samples extracted from the aging dataset [GSE:40279], the distribution of SD in ß value was plotted for the subsets of probes as described in (**B**). Numbers in brackets indicate KS statistics in comparison to the distribution of all probes. (**D**) DNAm at probe cg06961873 across 12 individuals exemplified the trichotomous pattern of DNAm hypothesized at a target CpG SNP_._ The three distinct levels of DNAm corresponded to sample genotype at SNP rs61775206, located at the target CpG: TT genotypes were assessed as hypomethylated, TC genotypes as approximately 50% methylated and CC genotypes as close to fully methylated. 450 k, Infinium HumanMethylation450 BeadChip; DNAm, DNA methylation.

Using the database of single nucleotide polymorphisms (dbSNP, National Center for Biotechnology Information (NCBI), Bethesda, MD, USA), one or more SNPs were annotated at 4.3% (n = 20,869) of target CpGs (Figure 1A). Most of these probes had only one target CpG SNP (n = 20,270), however, 599 had two or more (Additional file 2); 32.5% of probes with a target CpG SNP were not documented as variable by dbSNP, while 43.2% had a heterozygosity greater than 0.1 (Figure 1A). Being more frequent in the population, this second group of SNPs is more likely to affect the assessment of DNAm. The majority (67.3%) of the rs numbers for probes with target CpG SNPs corresponded to those annotated by Illumina as a SNP <10 bp. Differences between the annotations may be a result of our inclusion of SNPs in the C or G of the target CpG (whereas Illumina only annotated SNPs in the probe sequence, see Additional file 1), updates to the dbSNP database and the possibility that Illumina used a minimum heterozygosity as SNP inclusion criteria.

Theoretically, a bi- or tri-modal distribution of DNAm would be produced by a probe affected by sample genotypes at a target CpG SNP and this pattern would result in a high within-tissue SD in ß value (450 k array measure of DNAm ranging from 0 to 1). Thus, we examined the distribution of within-tissue SD in ß (n = 4/tissue) at probes annotated with a target CpG SNP, SNP <10 bp (excluding those probes also annotated with a target CpG SNP) and SNP >10 bp (Figure 1B, results for blood). The distribution of SD in ß for probes annotated with a target CpG SNP was most different (*p*= 1.78 × 19^-15^) from that of all probes based on a Kolmogorov-Smirnov (KS) test for difference in distribution. This trend was illustrated by a shift in the density curve for SD in ß of probes with target CpG SNPs in comparison to the curve for SD in ß of all probes (Figure 1B). To ensure that this finding was not an artifact of our small sample size, we performed the same analysis using a larger, publically available dataset, Gene Expression Omnibus (GEO) [GSE:40279], that had investigated age-associated DNAm changes in the blood of 656 individuals (aged 19 to 101 years) [24]. We extracted the younger half of samples (n = 261, aged 19 to 61 years) for our analysis since this roughly covered the age of the blood samples in our study. In this larger dataset (referred to as the ‘aging dataset’), the distribution of SD in ß for probes annotated with a target CpG SNP also exhibited the largest difference in distribution from that of all probes (*p* = 1.22 × 10^-14^) based on a KS test (Figure 1C).

We next hypothesized that highly variable probes (defined as within-tissue SD in ß ≥0.25), were likely compromised by the presence of a target CpG SNP. There were 780 such probes in blood, 819 in buccal, 666 in chorionic villus samples and 480 in the aging dataset that met this criterion (Table 1). We did not expect the number of probes affected by SNPs to be the same across tissues since all samples were from different individuals and thus of different genotypes. Comparing these variably methylated probes to the SNP annotation, 85.0%, 81.6%, 72.7% and 92.5% were annotated with a target CpG SNP in blood, buccal, chorionic villus samples and the aging dataset, respectively (Table 1). Of the highly variable probes, only four in blood, two in buccal and two in chorionic villi overlapped with the sex-specific autosomal probes described in the next section, thus we do not believe that these large SDs were driven by sex differences in the data. No probes in the aging dataset met this criterion.

**Table 1:** The majority of highly variable probes were annotated with SNPs

**Tissue**				
**Highly variable probes**^**a**^	**Blood**	**Buccal**	**Chorionic villi**	**Aging dataset**
**Total**	780	819	666	480
**Annotated with target CpG SNP**	663 (85.0)	668 (81.6)	484 (72.7)	444 (92.5)

^a^Defined as within-tissue SD in ß ≥0.25. Number in brackets is percentage (%) of total/tissue.

To confirm that a target CpG SNP could affect DNAm, samples were genotyped at a probe (cg06961873) that had an annotated target CpG SNP and SD in ß ≥0.25 in all three tissues. As predicted, homozygous C samples were assessed as hypermethylated, heterozygotes were assessed as approximately 50% methylated and homozygous T samples as hypomethylated (Figure 1D). Although we were not able to genotype samples, a histogram of DNAm at this same CpG site across the 261 aging dataset samples showed the same trimodal pattern of DNAm (Additional file 3). Other examples of highly variable probes in the aging dataset also illustrate this pattern (Additional file 3).

Given the demonstrated potential to bias the call of DNAm, we suggest that probes with a target CpG SNP should be disregarded in most analyses of the 450 k array. At minimum, 450 k users should carefully check candidate probes against the target CpG SNP annotation in addition to a current SNP database, as more polymorphisms are likely to be identified and validated in coming years. Although we have used a straightforward example to illustrate how a target CpG SNP may confound the assessment of DNAm, effects may also be observed at SNPs within the remainder of the probe, that is, outside of the target CpG. For example, polymorphisms throughout the interval of hybridization have been shown to affect the binding of probes used in Illumina mRNA expression arrays [25], which have the same probe lengths as the 450 k array. Similar effects have also been observed in Affymetrix mRNA expression arrays (Affymetrix, Santa Clara, CA, USA), although these use shorter probes that might be more sensitive to sequence mismatches [26]. Additionally, several studies have recognized the heritability of DNAm through the genetic-epigenetic interaction of methylation-associated SNPs (mSNPs) [27,28], suggesting that some SNP-associated differences in DNAm may be true differences and not due to technical artifacts.

### 8 to 9% of probes mapped to more than one location *in silico*

An additional confounding feature of the Infinium arrays is that some probes potentially map to multiple locations in the genome [10]. Signals from these non-specific probes likely represent a combination of DNAm at more than one location. Using alignment to four different *in silico* bisulfite-treated genomes [10], we identified 11.2% (n = 15,125) of type I probes and 7.7% (n = 26,812) of type II probes on the 450 k array as non-specific (total of 8.6% of 450 k probes). While the number of cross-hybridization loci per probe ranged from 2 to 1615, the majority of non-specific probes cross-hybridized to between two and five locations (52.4% of type I non-specific probes and 65.2% of type II non-specific probes). Within non-specific probes, 600 were intended to target sex chromosomes but also mapped to autosomal chromosomes, while 11,412 were intended to target autosomal chromosomes but also mapped to sex chromosomes (Table 2). Other publications have described how this second group of probes may be problematic in studies assessing sex differences in DNAm on autosomes or in studies where male and female subjects are analyzed together [10,29]. Thus we included in our annotation whether each probe cross-hybridized to sex or autosomal chromosomes.

**Table 2:** **Location of *in silico *** **cross-hybridization of non-specific probes**

		**Intended probe target: auto chrs**	**Intended probe target: sex chrs**
	**Total on array**	**473,864**	**11,648**
Non-specific probes	Cross-hybridize only to auto chrs	29,178	371
	Cross-hybridize only to sex chrs	540	747
	Cross-hybridize to auto and sex chrs	10,872	229
	Total: cross-hybridize to sex chrs	11,412	976
	Total: cross-hybridize to auto chrs	40,050	600
	Total	40,590	1,347

Auto, autosomal; chrs, chromosomes.

In the aging dataset, after excluding sex chromosome probes, but not our annotated non-specific probes, we used a false discovery rate (FDR) and minimum difference in DNAm (Δß) between sexes to identify autosomal probes that were differentially methylated between males (n = 133) and females (n = 128). An FDR of <1% and minimum Δß of 10% identified 75 sex-specific autosomal probes of which 40% were annotated to cross-hybridize to the sex chromosomes (Additional file 4). Although some true sex differences in DNAm likely exist on the autosomes, this result indicates that many of the large autosomal sex differences in DNAm may be an artifact of probe design and likely actually represent sex-chromosome differences in DNAm. Depending on the research question, some investigators may choose to exclude all or a subset of non-specific probes prior to data analysis, while others may include them and follow-up candidate probe specificity before establishing conclusions.

Homologous gene families, duplicated genes or repetitive elements have been proposed as potential causes of *in silico* cross-hybridization of Infinium probes [10]. Thus, for all 450 k probes, we annotated the number of nucleotides at the intended site of hybridization that mapped to repetitive DNA based on RepeatMasker (http://www.repeatmasker.org; RepeatMasker, Institute for Systems Biology, Seattle, WA, USA) annotation in BLAT [30]. For 72,957 probes (15.0% of 450 k probes), more than half of the nucleotides in the probe (>25 bps) was targeted to repetitive DNA. We had annotated 19,731 of these repetitive probes as non-specific, which reflects their *in silico* cross-hybridization. Interestingly, for 24,847 specific probes (that is, mapped only to the intended target), the entire probe (50 bps) was in repetitive DNA. This group of specific repetitive probes might be exploited to assess DNAm of repetitive elements; of interest to studies investigating changes in DNAm in cancer or in association with environmental exposure [31,32].

### Comparing Illumina and HIL annotation of probes highlighted differences between CpG classification systems

As previously mentioned, the 450 k array includes probes designed to target UCSC CpG islands, as well as shores, shelves and non-island regions, which we refer to as the ‘sea’ [9] (Additional file 5A, see methods for class definitions). Alternative ‘HIL’ CpG classes (that is, high-density CpG island (HC), intermediate-density CpG island (IC) and non-island (LC)) provide a different criterion for probe annotation based on CpG enrichment. We expanded the 450 k annotation by categorizing probes into four HIL classes: 1) HC probes, 2) IC probes, 3) ICshore probes (regions of intermediate-density CpG island that border HCs) and 4) LC probes (Additional file 5B, see methods for class definitions) [16,18].

**Figure 2:**
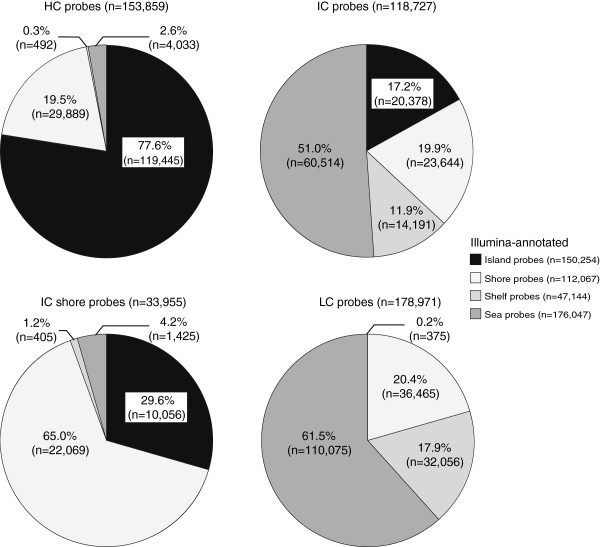
**Comparison of the genomic distribution of Illumina-annotated CpG probe classes within each HIL-annotated CpG probe class.** Within HCs, ICshores and LCs, the majority of probes were categorized into the respective Illumina-annotated CpG class. However, even though ICs and ICshores have the same CpG density, the distribution of probes based on Illumina CpG classes was different between these two HIL classes, suggesting a functional difference between ICs that border HCs and isolated ICs. HC, high-density CpG island; HIL, high-density CpG island (HC), intermediate-density CpG island (IC) and non-island (LC); ICshore, intermediate-density CpG island shore.

The distribution of probes within each Illumina-annotated CpG class was compared to the distribution of probes within each HIL-annotated CpG class (Additional file 6). The majority of probes were classified as anticipated, with 77.6% of HIL-annotated HC probes annotated as Illumina island probes, 65.0% of HIL-annotated ICshore probes annotated as Illumina shore probes and 61.5% of HIL-annotated LC probes annotated as Illumina sea probes (Figure 2). The largest difference in annotation was that close to half of HIL-annotated IC probes (51.0%) were Illumina-annotated sea probes, while the remainder of IC probes was distributed across Illumina-annotated islands (17.2%), shores (19.9%) and shelves (11.9%).

To elucidate potential functional differences between CpG classes, we examined the distribution of DNAm for both Illumina and HIL-annotated CpG classes (for blood, Additional file 7; buccal, Additional file 8 and chorionic villi, Additional file 9). Within each classification system, all distribution curves were significantly different from each other. On average, KS statistics were larger for comparisons between HIL CpG classes than for Illumina CpG classes (Additional files 7, 8, 9), indicative of more distinct distributions of DNAm in HIL CpG classes.

**Figure 3:**
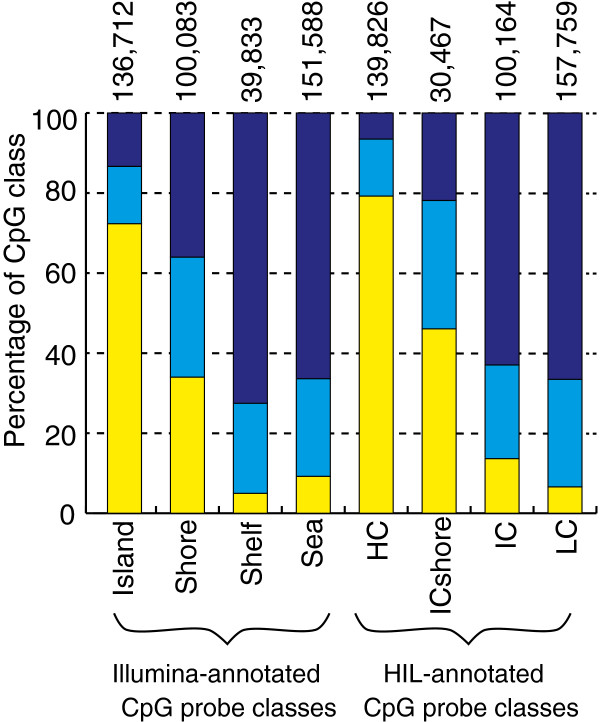
**Distinct patterns of DNAm within CpG classification systems.** Probes were grouped into three levels of DNAm based on average ß values within a tissue: hypomethylated (ß values of 0 to ≤0.2, yellow), heterogeneously methylated (ß values of >0.2 to <0.8, light blue) and hypermethylated (ß values of ≥0.8 to 1, dark blue). The percentage of probes in Illumina and HIL-annotated CpG classes was plotted for the three levels of DNAm in blood (n = 4). HIL CpG classes were more characteristic in their DNAm profiles than Illumina-annotated CpG classes. Numbers on top of bars indicate number of probes/class. DNAm, DNA methylation; HIL, high-density CpG island (HC), intermediate-density CpG island (IC) and non-island (LC); ICshore, intermediate-density CpG island shore.

Using blood as example, ß values were separated into three categories: hypomethylated (ß values of 0 to ≤0.2), heterogeneously methylated (ß values of >0.2 to <0.8) and hypermethylated (ß values of ≥0.8 to 1.0) (Figure 3 and Additional file 10) [7,33]. The majority of both HIL-annotated HC probes (79.2%) and Illumina-annotated island probes (72.3%) fell in the hypomethylated category in blood, consistent with the characteristic pattern of CpG island DNAm [33,34]. The distribution of DNAm within Illumina-annotated shore probes, HIL-annotated IC probes and HIL-annotated ICshore probes was different (for example, in the hypomethylated category 34.0%, 13.6% and 46.1%, respectively), suggesting that these CpG classes are distinctive. Interestingly, a higher proportion of Illumina-annotated shelf probes than Illumina-annotated sea probes were hypermethylated (72.6% vs 66.4% respectively), perhaps attributable to the differing CpG enrichment profile within shelves and seas (as demonstrated by the contribution of HIL-annotated HC, IC and LC probes to each of these classes, Additional file 6).

**Figure 4:**
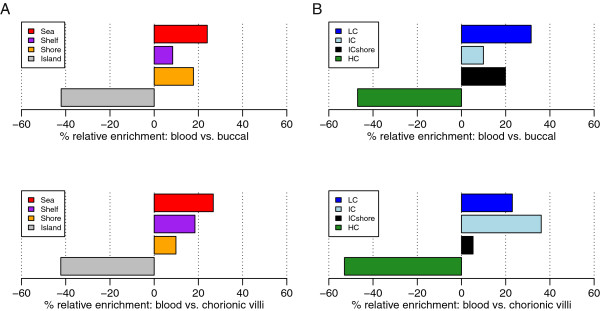
**Enrichment of differentially methylated probes in many CpG classes.** Probes that were differentially methylated between blood and buccal samples (n = 69,174), or between blood and chorionic villus samples (n = 91,255), were assessed for enrichment in (**A**) Illumina and (**B**) HIL-annotated CpG classes. Enrichment was plotted as ‘percentage relative enrichment’, representing the enrichment of tDM probes relative to the total percentage of probes within each CpG class. Negative percentage relative enrichment indicates that tDM probes were depleted in the given probe-type category whereas positive percentage relative enrichment indicates that tDM probes were enriched in the given probe-type category. All enrichment analyses were significant with the exception of ICshore probes in the comparison of blood versus chorionic villi. HIL, high-density CpG island (HC), intermediate-density CpG island (IC) and non-island (LC); ICshore, intermediate-density CpG island shore; tDM, differentially methylated between tissues.

Previous studies have shown that tissue-specific differences in DNAm occur in CpG island shores [19]. We were interested in assessing where tissue-specific differences in DNAm occur based on the Illumina versus HIL CpG classes. Thus, we examined probes that were differentially methylated between tissues (tDM) for enrichment within each CpG class. The highest number of tDM probes were identified between blood versus chorionic villus samples (91,255, 21.3% of probes), in comparison to chorionic villus versus buccal samples (75,021, 17.5% of probes) and blood versus buccal samples (69,174, 16.2% of probes). tDM probes were significantly depleted in Illumina-annotated islands and HIL-annotated HCs, and significantly enriched in all other CpG classes (Figure 4). Interestingly, the level of enrichment within each CpG class varied by the tissues compared (Figure 4 and Additional file 11).

A goal of the additional CpG classification of 450 k probes was to identify biologically-relevant structures that might underlie genome-wide changes in DNAm. The HIL CpG classes demonstrated a more extreme DNAm profile and larger percentage of tDM probes which may be reflective of biological processes. Intriguingly, even though ICs and ICshores have the same CpG density, distinct differences between these two classes emerged in our analyses, suggesting that ICs that border HCs are distinct from ICs on their own, highlighting the utility of this additional classification.

### DNAm was variable across nine gene feature groups

**Figure 5:**
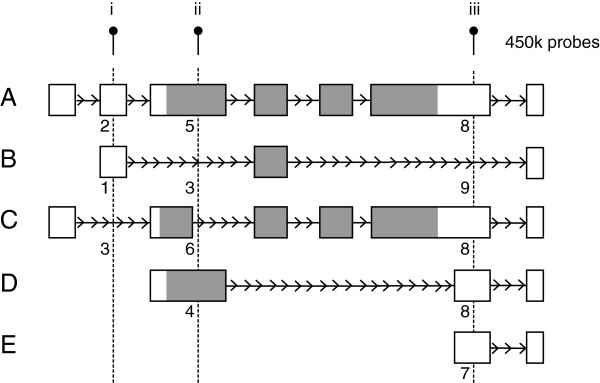
**Illustration of gene feature annotation.** Based on the overlap of three gene components (first exon vs exon vs intron) with three gene regions (5’UTR vs body vs 3’UTR) probes were annotated into the following nine gene feature groups: 1) 5’UTR first exons, 2) 5’UTR exons, 3) 5’UTR introns, 4) body first exons, 5) body exons, 6) body introns, 7) 3’UTR first exons, 8) 3’UTR exons and 9) 3’UTR introns (corresponding to numbers below transcripts). A given probe could be annotated with more than one gene feature, as illustrated by the multiple transcripts (**A** to **E**) of a fictional gene. Probe i would be annotated as 5’UTR exon, 5’UTR first exon and 5’UTR intron; probe ii would be annotated as body exon, 5’UTR intron, body intron, body first exon; and probe iii would be annotated as 3’UTR exon, 3’UTR intron, 3’UTR exon, 3’UTR exon and 3’UTR first exon. White boxes represent untranslated exons, grey boxes represent translated exons. 5’UTR, 5’ untranslated region; 3’UTR, 3’ untranslated region.

There is increasing evidence that DNAm of gene features outside of CpG islands and promoters may be an important marker of gene expression. For example, it has been shown that DNAm of the first exon is correlated with transcriptional repression [35]. Coverage of regions outside of CpG islands and promoters was dramatically increased from the 27 k to 450 k array, however Illumina only categorized probes into six gene feature groups: TSS1500 (within 1500 bps of a transcription start site (TSS)), TSS200 (within 200 bps of a TSS), 5’UTR (5’ untranslated region), first exon, body and 3’UTR (3’ untranslated region). Given the number of probes on the array, a more detailed gene structure classification might increase the potential to observe subtle biologically-relevant trends in DNAm. Thus we expanded on gene feature annotation by: 1) annotating the distance of each probe to the closest TSS and 2) classifying probes into nine groups based on three gene components (first exons, exons and introns) and three gene regions (5’UTR, body and 3’UTR). Probes were grouped into: 1) 5’UTR first exons, 2) 5’UTR exons, 3) 5’UTR introns, 4) body first exons, 5) body exons, 6) body introns, 7) 3’UTR first exons, 8) 3’UTR exons and 9) 3’UTR introns using a) transcript and b) RefGene name. Due to alternative TSS and splicing, a given probe could be categorized into several gene feature categories (Figure 5).

**Figure 6:**
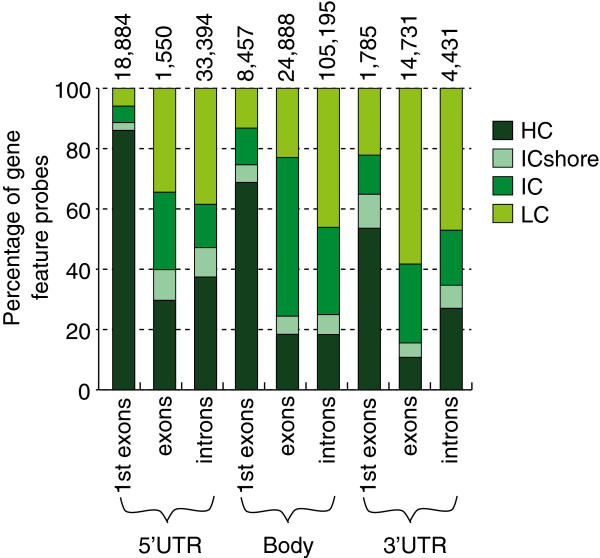
**Contribution of HIL CpG classes to probes in nine gene feature groups.** The percentage of probes within each HIL CpG class was different for each gene feature group. Numbers on top of bars indicate the number of probes/gene feature group; a total of 213,315 probes were located within these nine gene feature groups. HIL, high-density CpG island (HC), intermediate-density CpG island (IC) and non-island (LC); ICshore, intermediate-density CpG island shore.

**Figure 7:**
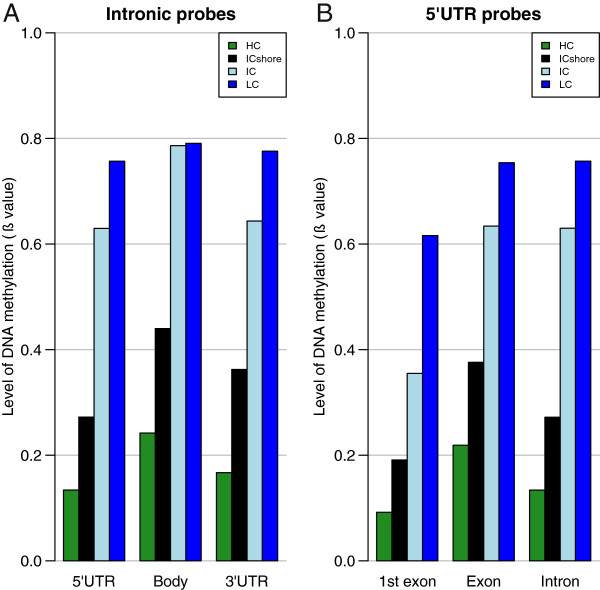
**Variation of gene feature DNAm within a CpG class.** The level of DNAm was plotted as an average ß value for each gene feature in blood. Analyses were conducted within each HIL CpG class due to the large differences in DNAm that were observed between classes. Average ß values varied across probes by (**A**) gene location, as exemplified by intronic probes and (**B**) gene components, as exemplified by 5’UTR probes. 5’UTR, 5’ untranslated region; DNAm, DNA methylation; HIL, high-density CpG island (HC), intermediate-density CpG island (IC) and non-island (LC); ICshore, intermediate-density CpG island shore.

Due to the observed differences in DNAm across HIL CpG classes detailed in the previous section, gene features were further subclassified by HIL CpG class. Given the known bias in the distribution of CpGs in the genome [14], there was a predictable unequal distribution of the proportion of probes annotated to each HIL CpG class across gene feature groups (Figure 6 and Additional file 12). For example, HC probes were significantly overrepresented in first exons found in the 5’UTR and gene body, while LC probes were significantly underrepresented in both these groups. Within each HIL CpG class, trends in DNAm across gene features were consistent (Additional file 12). For example, in blood, DNAm of intronic probes increased from 5’UTR to 3’UTR to gene body probes (Figure 7A), while DNAm of 5’UTR probes increased from first exon to intron to exon probes (Figure 7B).

We were also interested in assessing where tissue-specific differences in DNAm occurred based on gene features. Thus, we examined tDM probes for enrichment within each gene feature group (again separated by CpG class, Additional file 13). tDM probes in first exons were significantly depleted in 5’UTRs located in HCs and ICshores, but significantly enriched in 5’UTRs located in LCs. HC exons were significantly enriched for tDM probes in 5’UTR, body and 3’UTR across all tissue comparisons, perhaps due to biological significance or small probe numbers in these categories. Although CpG classes were primarily associated with differences in DNAm, gene structure is also an important factor to consider when analyzing 450 k array results.

## Conclusion

With the advent of next-generation sequencing applied to bisulfite converted samples, measurement of DNAm will truly be possible on a genome-wide, sequence-specific scale. However, difficulties currently lie in the alignment of reduced complexity reads as well as biologically-relevant interpretation of data [36]. Array-based technologies, which target specific genomic regions of interest, are of value for assessing physiologically-relevant changes in studies of human health and disease. Detailed and comprehensive annotation of locus-specific arrays is paramount to successful analysis and interpretation of data.

In this article, we presented an expanded annotation of the 450 k array including both compromised probe annotation and additional biologically-relevant annotation. Our expanded annotation has been deposited as a platform on the NCBI GEO (http://www.ncbi.nlm.nih.gov/geo) under the accession [GSE:42409]. Based on our findings, we suggest that all 450 k users analyze data with the following factors in mind. Probe signals may be biased by the presence of SNPs in the target CpG and/or binding of probes to multiple genomic locations. SNPs at the target CpG may be especially problematic in studies with small sample size, as chance may result in dramatic differences in the frequency of polymorphisms between groups. However, false positives may still result in studies with larger sample sizes, if groups are not ethnically matched. Additionally, DNAm patterns within CpG enrichment classes or gene features could overshadow findings between study groups if probes are not separated and considered within these genomic features.

There are certainly other methods and filters that can be applied to 450 k array analyses that were not touched upon in this article. Notably, a recent study excluded 450 k probes that mapped to copy-number variations (CNVs) because of the potential to bias measurement of DNAm [37]. Furthermore these authors set a criterion of ‘comethylation’ to identify differentially methylated regions, that is, all probes within a 250 bp window had to show the same trend in DNAm. As more studies using the 450 k array are published, we will undoubtedly see various combinations of applied filters and methodological practices for data analysis. In this era of extensive data collection using such high-throughput assays, it is vital that the type of biological sample as well as the research question is carefully considered in relation to downstream analytical choices as well as the technical platform.

## Methods

### Annotation

To complete the expanded annotation, we calculated additional probe location information based on the Illumina-provided MAPINFO GenomeStudio column (location of the C in the target CpG): 1) the interval of the target CpG (CpG), 2) the interval containing the probe but excluding the target CpG (Probe_w/o CpG_) and 3) the interval of the entire probe (entire probe) (Additional files 1 and 14). Probe type (type I vs type II) and strand of design (F or R) were taken into consideration when calculating genomic location. Ten type I and ten type II probes were manually checked against the annotated probe sequence. A UCSC track was created containing the targeted Cs on the 450 k array (Additional file 15). All of the annotation and analysis of the expanded annotation was conducted on 485,512 probes, including both cg (CpG loci) and ch (non-CpG loci) probes but excluding rs (SNP assay) probes, unless otherwise specified.

### SNP annotation

The dbSNP131 table was imported into Galaxy (http://galaxyproject.org; Galaxy, Pennsylvania State University, PA, USA) from UCSC [38]. Only rs numbers for SNPs that were an interval of 1 bp in length and of the highest quality (weight = 1) were included in the annotation. An interval file was uploaded into Galaxy using the hg19 location we annotated for the interval of each probe spanning the C and G of the target CpG for cg probes only. The probe file was intersected with the dbSNP131 table to create a list of probes with documented SNPs in the C or G of the target CpG (target CpG SNP). This file was collapsed in R (http://www.r-project.org; R Foundation for Statistical Computing, Vienna University of Economics and Business, Vienna, Austria) to create a list of rs numbers for each probe, since some target CpGs were documented with more than one SNP. The rs numbers for SNPs in the target CpG were included in the expanded annotation in the ‘target CpG SNP’ column (n = 20,270), while the number of SNPs/probes was annotated in the ‘n_target CpG SNP’ column.

### Non-specific probe annotation

To identify probes that potentially have multiple genomic targets (non-specific probes), we followed the method described by Chen *et al.*[10]. Special treatment of type II probes was required as the Illumina annotation has noted Cs in CpGs within the probe as an ‘R’ SNP. For type II probes that contained Rs we considered two probe sequence versions, one with all Rs replaced by As and the other with all Rs replaced by Gs. Using these conditions, we matched each of the 450 k probes with the Illumina-annotated genomic location (intended target).

Briefly, we used BLAT [30] to align probe sequences to four versions of the hg19 draft sequence genome: 1) a fully unmethylated ‘bisulfite treated’ genome, with all Cs converted to Ts; 2) a fully methylated ‘bisulfite treated’ genome, with only non-CpG Cs converted to Ts; 3) and 4) were the above treatments on the reverse complement sequence. BLAT was run using the following parameters: stepSize = 5, wordsize = 11 and repMatch = 1,000,000; lowering the word length led to only fractionally more hits. The selection criterion used was as previously outlined: for a probe to be considered non-specific, there had to be 90% identity over the aligned region, at least 40 of 50 matching bps, no gaps, and the 50^th^ nucleotide had to align, as the probe hybridizes to the target CpG at this position [10]. The number of non-specific probes hits were annotated in the expanded annotation ‘AlleleA_Hits, AlleleB_Hits’ columns, while the site of cross-hybridization was annotated in the columns ‘XY_Hits’ (if at least one hit was on a sex chromosome) and ‘Autosomal_Hits’ (if at least one hit was on an autosomal chromosome). Repetitive sequences from RepeatMasker were marked in lowercase in the four genomes. Thus we identified the amount of repetitive DNA within the Illumina-intended alignment of each probe in the expanded annotation column ‘n_bp_repetitive’.

### CpG enrichment annotation

Illumina categorized probes in CpG islands (GenomeStudio column ‘Relation_to_UCSC_CpG_Island’) based on the UCSC Genome Browser criteria of CG content >50%, Obs/Exp CpG ratio >0.60 and length >200 bps. Shores and shelves were identified based on their relationship to a CpG island; shores as the 2 kbs up- and down-stream of CpG islands and shelves as the 2 kbs outside of shores. The remaining probes were located in non-island regions, which we refer to as the ‘sea’ [9] (Additional file 5A).

We annotated probes into four HIL CpG classes based on alternative CpG enrichment criteria: high-density CpG island probes (HC, n = 153,859), intermediate-density CpG island probes (IC, n = 118,727), ICshore probes (probes in ICs that border HCs, n = 33,955) and non-island probes (LC, n = 178,971) (Additional file 5B). This annotation has been added in the ‘HIL_CpG_class’ column of the expanded annotation. To locate probes within each of the four CpG classes, we first annotated these CpG enrichment classes throughout the genome. The hg19 genomic sequence was downloaded from UCSC in overlapping segments and read by CpGIE, a Java software program [39]. CpGIE searches input sequences in sliding windows based on user-set criteria. HCs were defined as regions with CG content >55%, Obs/Exp CpG ratio >0.75 and length >500 bps, while ICs were defined as regions with CG content >50%, Obs/Exp CpG ratio >0.48 and length >200 bps [16,18]. CpGIE HC and IC output was merged into a single file for each chromosome, duplicate islands were removed and CpG islands were identified as follows: ICs, isolated regions of the genome with IC density; ICshores, regions of the genome with IC density that were next to regions with HC density; HCs, any region of the genome with HC density; and LCs, regions that were not of IC or HC density. Islands were given unique names in the annotation, for example, chr8_IC:49890018–49891221 (chr#_CpG class: genomic start–genomic end). The hg19 HC and IC islands have been complied into a UCSC track available in Additional file 16. The hg19 HIL annotation was intersected with the genomic location (hg19) of 450 k targets in Galaxy to assign probes into the four CpG classes. An annotation of probes into HIL CpG islands using the detailed nomenclature can be found in the expanded annotation column ‘HIL_CpG_Island_Name’.

### Gene feature and TSS annotation

Using the NCBI Reference Sequence (RefSeq) gene annotation, we annotated probes into nine groups based on three gene components (first exons, exons and introns) and three gene regions (5’UTR, body and 3’UTR). Probes were grouped into: 1) 5’UTR first exons, 2) 5’UTR exons, 3) 5’UTR introns, 4) body first exons, 5) body exons, 6) body introns, 7) 3’UTR first exons, 8) 3’UTR exons and 9) 3’UTR introns (Figure 5). Briefly, the hg19 RefSeq table was downloaded from UCSC [38]. Exon and intron information was extracted and parsed into genomic interval data with the most upstream exon denoted as the first exon. Next, 5’UTR, gene body and 3’UTR location was parsed into genomic interval data utilizing the transcription start/stop and coding start/stop information from RefSeq. Intersection was performed between each of 5’UTR, gene body and 3’UTR with first exon, exon and intron intervals to generate the nine gene features. The gene feature intervals were then intersected with the hg19 genomic location of 450 k targets in R to assign probes into the nine gene features. This annotation was completed using both RefSeq gene names and transcript names. Gene feature annotation was conducted using the GenomicRanges package in R [40].

The hg19 UCSC knownGene table [38] was downloaded to Galaxy and the closest TSS for each probe was annotated, regardless of whether the probe was located within the same gene. For each probe, the distance to the closest TSS, gene name and transcript name was noted in the expanded annotation columns ‘Closest_TSS’, ‘Distance_closest_TSS’, ‘Closest_TSS_gene_name’ and ‘Closest_TSS_Transcript’.

### Sample collection

Two male and two female chorionic villus samples were collected through the BC Women’s Hospital & Health Centre, Vancouver, BC, Canada, as controls for a study of chromosomal abnormalities in the placenta. DNA was extracted from a small piece of chorionic villi as previously described [41]. For each placental sample (n = 4), DNA from two independent chorionic villi was combined in equal amounts prior to bisulfite conversion to ensure a representative sample of the placenta. DNA was extracted by standard salt method. Two male and two female blood samples were collected as adult controls for ongoing studies of respiratory disease and epigenetics (n = 4). Peripheral blood mononuclear cell (PBMC) DNA was extracted according to standard procedures. Buccal epithelial samples were collected from two males and two females for a study on maternal care effects on childhood DNAm (n = 4). Buccal samples were collected using Isohelix DNA Buccal Swabs (Cell Projects Ltd, Harrietsham, Kent, UK), and stabilization reagents and DNA were extracted using Isohelix DNA Isolation Kits (Cell Projects Ltd) as per the manufacturer’s protocols.

### Illumina 450 k array

Two ug of genomic DNA was purified using the DNeasy Blood & Tissue Kit (Qiagen, Valencia, CA, USA) following the manufacturer’s protocol. Purified DNA quality and concentration were assessed with a NanoDrop ND-1000 (Thermo Scientific, Waltham, MA, USA) prior to bisulfite conversion. One ug of purified genomic DNA was bisulfite converted using the EZ DNA Methylation Kit (Zymo Research, Orange, CA, USA) following the manufacturer’s protocol. Bisulfite DNA quality and concentration were assessed using the NanoDrop and, if required, samples were concentrated to approximately 50 ng/ul using a SpeedVac (Thermo Electron Corporation, Waltham, MA, USA). Following the Illumina 450 k array protocol, 4 ul of bisulfite converted sample was whole-genome amplified, enzymatically digested, hybridized to the array and then single nucleotide extension was performed [9].

Two assay types are used by the 450 k array to measure DNAm: Infinium I (type I probes) and Infinium II (type II probes), bound to beads scattered throughout the array. When a probe successfully binds to DNA, a single fluorescently labeled nucleotide extends off the probe and this signal is read by an Illumina scanner. The Infinium I assay uses two bead types specific to the CpG of interest: an unmethylated (u) and a methylated (m) bead, each with a different probe design (ProbeA (u) and ProbeB (m)). Both type I probes for a given CpG fluoresce in the same color channel (either red (Cy5) or green (Cy3)). The Infinium II assay uses only one bead type for each CpG of interest, an m + u bead. One probe is designed for each type II target site and the color of fluorescence is based on which nucleotide is incorporated in the single base extension step. The incorporation of an A or T signals an unmethylated site in red (u) and the incorporation of a C or a G signals a methylated site in green (m) [8].

Chips were scanned using an Illumina HiScan on a two-color channel to detect Cy3 labeled probes on the green channel and Cy5 labeled probe on the red channel. Illumina GenomeStudio Software 2011.1 was used to read the array output and conduct background normalization. The signalA, signalB and probe intensity were exported for autosomal probes and read into R. M values were generated using the Bioconductor (http://www.bioconductor.org; Fred Hutchinson Cancer Research Center, Seattle, WA, USA) methylumi package, M = log_2_(intensity m + 1/intensity u + 1) since this value has been shown to be valid for statistical analyses [42]. Following correction for chip to chip color bias using the Bioconductor lumi package [43] and probe type correction using subset-quantile within array normalization (SWAN) [44], M values were converted to ß values using the equation ß = (2^M^/(2^M^ + 1)). The ß value is a number ranging from 0 to 1 that is directly proportional to percentage DNAm; thus to ease interpretation, we have reported results as ß values. The microarray data used in this article was submitted to the NCBI GEO under accession number [GSE:42409]. Probes with a detection *p* value >0.01 in any sample, probes with no ß value in any sample, all rs and ch probes, all sex chromosome and non-specific probes were removed prior to analyses. The level of DNAm for 428,216 probes in our sample dataset was intersected with the expanded annotation for further analyses.

### Processing of aging dataset

Series matrix files were downloaded for [GSE:40279] containing ß values for 473,039 probes per sample [24]. We worked with the subset of samples that roughly matched the age of the samples used in our study (n = 261, aged 19 to 61 years). Probes with no ß value in any sample, all sex chromosome probes, all rs and all ch probes were removed from the dataset. For SNP analyses, non-specific probes were also removed, however these were retained in the analysis of autosomal sex-specific probes. For the discovery of autosomal probes with sex differences in DNAm, ß values were read into R, converted into M values using the Bioconductor package lumi [43] and then significance analysis of microarrays (SAM) was conducted using the Bioconductor package siggenes [45]. At FDR <1%, 10,139 autosomal probes were identified as significantly different between male and female samples. Next, this list was crossed with a list of Δß values for each probe calculated by taking the absolute value of the difference between average ß of males and average ß of females.

### Pyrosequencing

Probe cg06961873 was selected for genotype validation of SNP rs61775206 in each sample. Primers were designed using PSQ Assay Design software version 1.0.6 (Biotage AB, Uppsala, Sweden). Primer sequences and probe information are available in Additional file 17. Using the following conditions, 0.5 ul of genomic DNA was PCR-amplified: 95°C for 5 minutes, (95°C for 20 seconds, 55°C for 20 seconds, 75°C for 20 seconds) × 50, 72°C for 5 minutes. Genotyping was performed using a PyroMark MD system (Biotage AB) and analyzed with PSQ 96MA SNP software (Biotage AB).

### Statistical analyses

A KS test was used to assess the difference in distribution of SD in ß values for probes that contained SNPs. The KS statistic represents the maximum absolute difference between the cumulative distributions of two functions. Probes with small within-tissue SD in ß (<0.10) were removed from all probe groups to increase the power of the analysis. Probes with a target CpG SNP were removed from the SNP <10 bp group. The number of probes included in the SD in ß distribution curves for blood samples was 5,450 for all probes, 809 for SNP >10 bp, 402 for SNP <10 bp and 2,190 for target CpG SNP, and for the aging dataset was 6,267 for all probes, 1,022 for SNP >10 bp, 362 for SNP <10 bp and 2,753 for target CpG SNP. KS tests were also used to assess the difference in distribution of DNAm between Illumina CpG classes and between HIL CpG classes. Fisher’s exact test was used to compare the distribution of the number of probes within the three levels of DNAm for both Illumina and HIL-annotated CpG classes: hypomethylated (ß values of 0 to ≤0.2), heterogeneously methylated (ß values of >0.2 to <0.8) and hypermethylated (ß values of ≥0.8 to 1.0). Enrichment analyses of tDM probes were performed in Python (Python Software Foundation). To select tDM probes, DNAm was first averaged for each probe within a tissue. A z-score was calculated for each probe comparison between tissues. A *p* value cutoff of 0.05 was selected with a Bonferroni correction to account for repeated comparisons [19]. KS and Fisher’s exact tests were performed in R. Statistical significance was considered as tests with *p* values <1.0 × 10^-7^. All figures were created in R and Adobe Illustrator CS6.

## Abbreviations

27 k: Infinium HumanMethylation27k BeadChip; 3’UTR: 3’ untranslated region; 450 k: Infinium HumanMethylation450 BeadChip; 5’UTR: 5’ untranslated region; auto: autosomal; bp: base pair; chrs: chromosomes; CNV: copy-number variation; dbSNP: database of single nucleotide polymorphisms; DNAm: DNA methylation; EWAS: epigenome-wide association studies; FDR: false discovery rate; GEO: Gene Expression Omnibus; GWAS: genome-wide association studies; HC: high-density CpG island; HIL: high-density CpG island (HC), intermediate-density CpG island (IC) and non-island (LC) or low-density regions; IC: intermediate-density CpG island; ICshore: intermediate-density CpG island shore; KS: Kolmogorov-Smirnov; LC: non-island; mSNP: methylation-associated SNP; NCBI: National Center for Biotechnology Information; Obs/Exp: Observed/Expected; PBMC: peripheral blood mononuclear cell; PCR: polymerase chain reaction; RefSeq: Reference Sequence; SAM: significance analysis of microarrays; SNP: single nucleotide polymorphism; SWAN: subset-quantile within array normalization; tDM: differentially methylated between tissues; TSS: transcription start site; UCSC: University of California, Santa Cruz.

## Competing interests

The authors declare that they have no competing interests.

## Authors’ contributions

EMP carried out Pyrosequencing, conducted SNP annotation and analysis, participated in CpG island annotation and analysis, participated in the design of the study, and drafted the manuscript. AMC participated in CpG island annotation and analysis, participated in the design and analysis of the study, and conceived of the study. LLL processed Illumina data, conducted gene feature annotation and participated in the design of the study. PF conducted enrichment analyses. EE conducted non-specific probe analyses and participated in the design of the study. MSK and CJB conceived of the study, participated in data analysis and participated in the design of the study. WPR participated in data analysis and design of the study. All authors contributed to the writing of the manuscript, and read and approved the final version.

## Supplementary Material

Additional file 1 **Relative location of probes to target CpG.** To complete our analysis, it was necessary to locate 450 k probes within the genome. Illumina annotated the hg19 location of each target C (called mapinfo) and the strand on which the probe was designed; R probes bind to the negative strand, whereas F probes bind to the positive strand. With this information we annotated the start and end coordinates for all probes on the array. Refer to Additional file 14 for the start and end location for each probe type. Type I versus type II probes and F versus R probes align differently with target CpGs. Single nucleotide extension of a probe occurs by one of four fluorescently labeled nucleotides, A and T are labeled in red, while C and G are labeled in green. The color of single nucleotide extension of type I probes is not dependant on whether the target site is methylated or unmethylated; however, for type II probes, incorporation of an A or T signals an unmethylated site in red and the incorporation of a C or a G signals a methylated site in green. Click here for file

Additional file 2 Frequency of target SNP CpG/probe. Click here for file

Additional file 3 **Distribution of DNAm at three highly variable probes.** The level of DNAm was plotted for three highly variable probes (SD in ß ≥0.25) annotated with a target CpG SNP, across the 261 individuals in the aging dataset. cg06961873 corresponds to the CpG site genotyped in Figure 1D. A trimodal pattern of DNAm was observed at these three exemplary sites, indicating that DNAm measured at these sites may reflect sample genotype. Click here for file

Additional file 4 List of autosomal probes with sex differences in DNAm. Click here for file

Additional file 5 **Illustration of Illumina and HIL CpG classes.** (**A**) Diagram of Illumina-annotated probes, based on their relative location to a CpG island: within the island, shore or shelf. We used the term ‘sea probes’ to refer to probes that were not annotated into one of the Illumina CpG classes. Islands were defined based on UCSC criteria: CG content >50%, Obs/Exp, CpG ratio >0.60 and length >200 bps. Shores were defined as the 2 kb up- and down-stream of a CpG island and shelves as the 2 kb outside of a shore. (**B**) The HIL definition of CpG islands was used to annotate probes into three CpG classes: HC probes (map to a high-density CpG island or HC), IC probes (map to an isolated intermediate-density CpG island or IC) and ICshore probes (map to a region with IC density that borders an HC). The remainder of probes did not map to a CpG island and were thus considered non-island or LC probes. HCs were defined as CG content >55%, Obs/Exp CpG ratio >0.75 and length >500 bps, while ICs were defined as CG content >50%, Obs/Exp CpG ratio >0.48 and length >200 bps. Click here for file

Additional file 6 Distribution of probes within Illumina-annotated and HIL-annotated CpG classes. Click here for file

Additional file 7 **Distinct patterns of DNAm across Illumina-annotated and HIL-annotated CpG classes in blood.** Density curves were plotted using average ß values for probes within each Illumina-annotated and HIL-annotated CpG class in blood (n = 4). The number of probes contributing to each curve was: island = 136,712, shore = 100,083, shelf = 39,833, sea = 151,588, HC = 139,826, ICshore = 100,164, IC = 30,467 and LC = 157,759. For Illumina-annotated CpG classes, KS statistics in comparison to the distribution of DNAm of sea probes was 0.67 for island probes, 0.34 for shore probes and 0.06 for shelf probes. For HIL-annotated CpG classes, KS statistics in comparison to the distribution of DNAm of LC probes was 0.77 for HC probes, 0.53 for ICshore probes and 0.08 for IC probes. Click here for file

Additional file 8 **Distinct patterns of DNAm across Illumina-annotated and HIL-annotated CpG classes in buccal samples.** Density curves were plotted using average ß values for probes within each Illumina-annotated and HIL-annotated CpG class in buccal samples (n = 4). The number of probes contributing to each curve was: island = 136,712, shore = 100,083, shelf = 39,833, sea = 151,588, HC = 139,826, ICshore = 100,164, IC = 30,467 and LC = 157,759. For Illumina-annotated CpG classes, KS statistics in comparison to the distribution of DNAm of sea probes was 0.66 for island probes, 0.32 for shore probes and 0.06 for shelf probes. For HIL-annotated CpG classes, KS statistics in comparison to the distribution of DNAm of LC probes was 0.76 for HC probes, 0.49 for ICshore probes and 0.07 for IC probes. Click here for file

Additional file 9 **Distinct patterns of DNAm across Illumina-annotated and HIL-annotated CpG classes in chorionic villi.** Density curves were plotted using average ß values for probes within each Illumina-annotated and HIL-annotated CpG class in chorionic villi (n = 4). The number of probes contributing to each curve was: island = 136,712, shore = 100,083, shelf = 39,833, sea = 151,588, HC = 139,826, ICshore = 100,164, IC = 30,467 and LC = 157,759. For Illumina-annotated CpG classes, KS statistics in comparison to the distribution of DNAm of sea probes was 0.61 for island probes, 0.28 for shore probes and 0.08 for shelf probes. For HIL-annotated CpG classes, KS statistics in comparison to the distribution of DNAm of LC probes was 0.72 for HC probes, 0.45 for ICshore probes and 0.10 for IC probes. Click here for file

Additional file 10 Distribution of ß values within Illumina-annotated and HIL-annotated CpG classes for blood. Click here for file

Additional file 11 Enrichment of differentially methylated probes in Illumina-annotated and HIL-annotated CpG classes. Click here for file

Additional file 12 Average DNAm and SD of nine gene features. Click here for file

Additional file 13 Enrichment of tDM probes within gene features. Click here for file

Additional file 14 Calculation of intended genomic location of 450k probes. Click here for file

Additional file 15 UCSC track of 450 k target Cs. Click here for file

Additional file 16 UCSC track of HC/IC CpG islands. Click here for file

Additional file 17 Primers for genotyping validation of a target CpG SNP. Click here for file
