# Supplementary figures and images for: Additional annotation enhances potential for biologically-relevant analysis of the Illumina Infinium HumanMethylation450 BeadChip array
Source: Epigenetics Chromatin. 2013 Mar 3;6:4. doi: 10.1186/1756-8935-6-4 (PMC3740789; doi:10.1186/1756-8935-6-4)

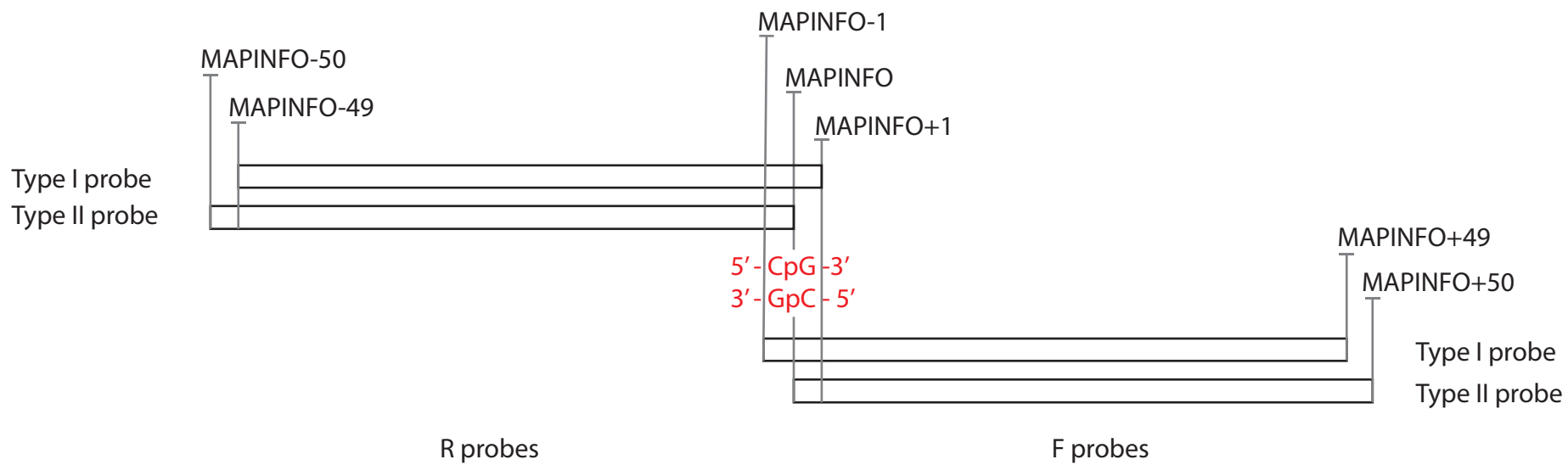

Supplement: Additional file 1 — Relative location of probes to target CpG. To complete our analysis, it was necessary to locate 450 k probes within the genome. Illumina annotated the hg19 location of each target C (called mapinfo) and the strand on which the probe was designed; R probes bind to the negative strand, whereas F probes bind to the positive strand. With this information we annotated the start and end coordinates for all probes on the array. Refer to Additional file 14 for the start and end location for each probe type. Type I versus type II probes and F versus R probes align differently with target CpGs. Single nucleotide extension of a probe occurs by one of four fluorescently labeled nucleotides, A and T are labeled in red, while C and G are labeled in green. The color of single nucleotide extension of type I probes is not dependant on whether the target site is methylated or unmethylated; however, for type II probes, incorporation of an A or T signals an unmethylated site in red and the incorporation of a C or a G signals a methylated site in green. [file 1756-8935-6-4-S1.pdf]

# Distribution of DNA methylation

**cg06961873**

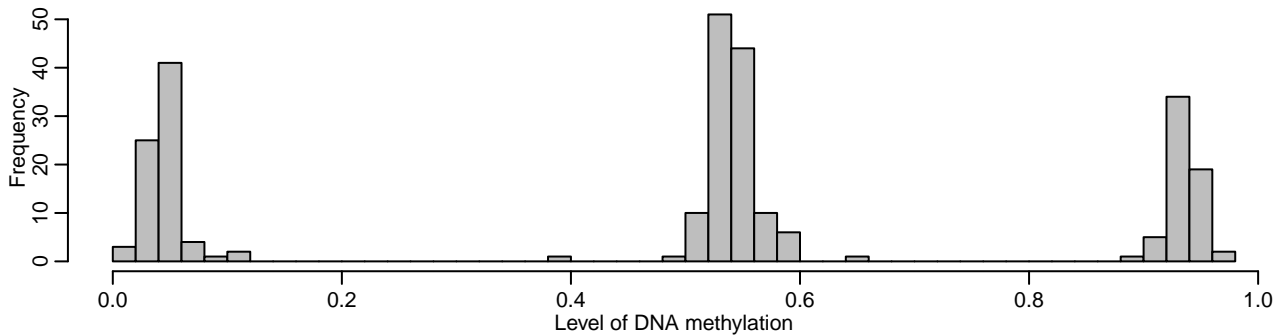

**cg25593194**

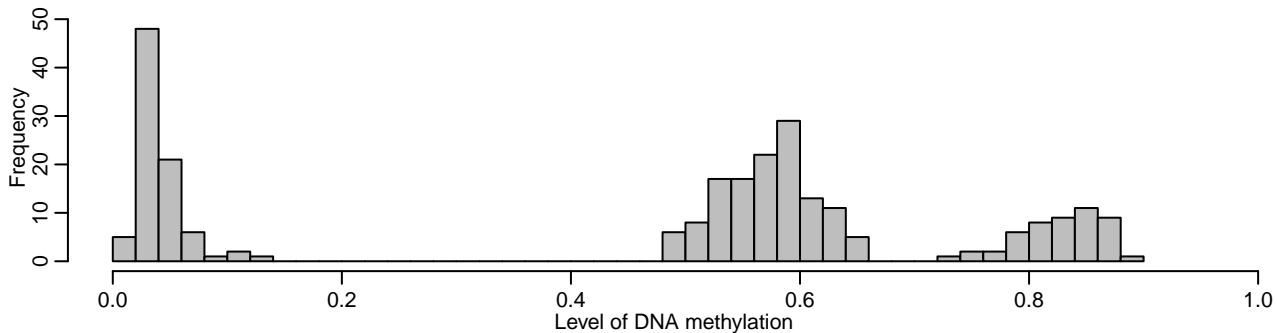

**cg16908938**

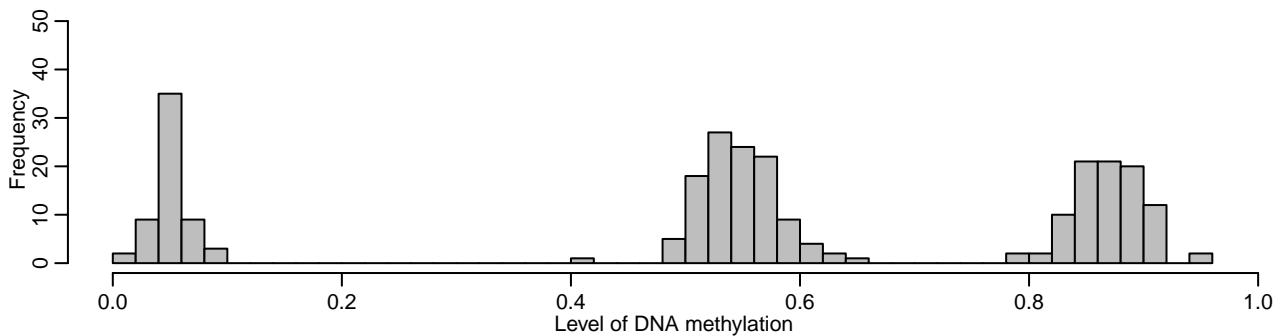

Supplement: Additional file 3 — Distribution of DNAm at three highly variable probes. The level of DNAm was plotted for three highly variable probes (SD in ß ≥0.25) annotated with a target CpG SNP, across the 261 individuals in the aging dataset. cg06961873 corresponds to the CpG site genotyped in Figure 1D. A trimodal pattern of DNAm was observed at these three exemplary sites, indicating that DNAm measured at these sites may reflect sample genotype. [file 1756-8935-6-4-S3.pdf]

A

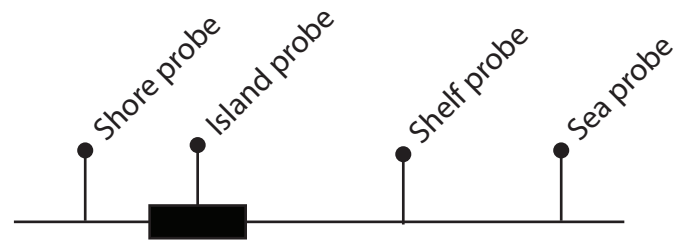

B

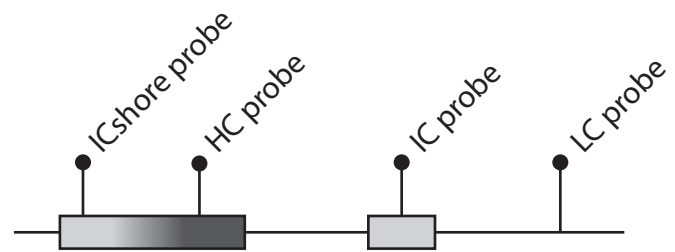

■ UCSC Island

■ Intermediate density CpG island (IC)

■ High density CpG island (HC)

Supplement: Additional file 5 — Illustration of Illumina and HIL CpG classes. (A) Diagram of Illumina-annotated probes, based on their relative location to a CpG island: within the island, shore or shelf. We used the term ‘sea probes’ to refer to probes that were not annotated into one of the Illumina CpG classes. Islands were defined based on UCSC criteria: CG content >50%, Obs/Exp, CpG ratio >0.60 and length >200 bps. Shores were defined as the 2 kb up- and down-stream of a CpG island and shelves as the 2 kb outside of a shore. (B) The HIL definition of CpG islands was used to annotate probes into three CpG classes: HC probes (map to a high-density CpG island or HC), IC probes (map to an isolated intermediate-density CpG island or IC) and ICshore probes (map to a region with IC density that borders an HC). The remainder of probes did not map to a CpG island and were thus considered non-island or LC probes. HCs were defined as CG content >55%, Obs/Exp CpG ratio >0.75 and length >500 bps, while ICs were defined as CG content >50%, Obs/Exp CpG ratio >0.48 and length >200 bps. [file 1756-8935-6-4-S5.pdf]

# Distribution of DNA methylation in blood

## Illumina CpG classes

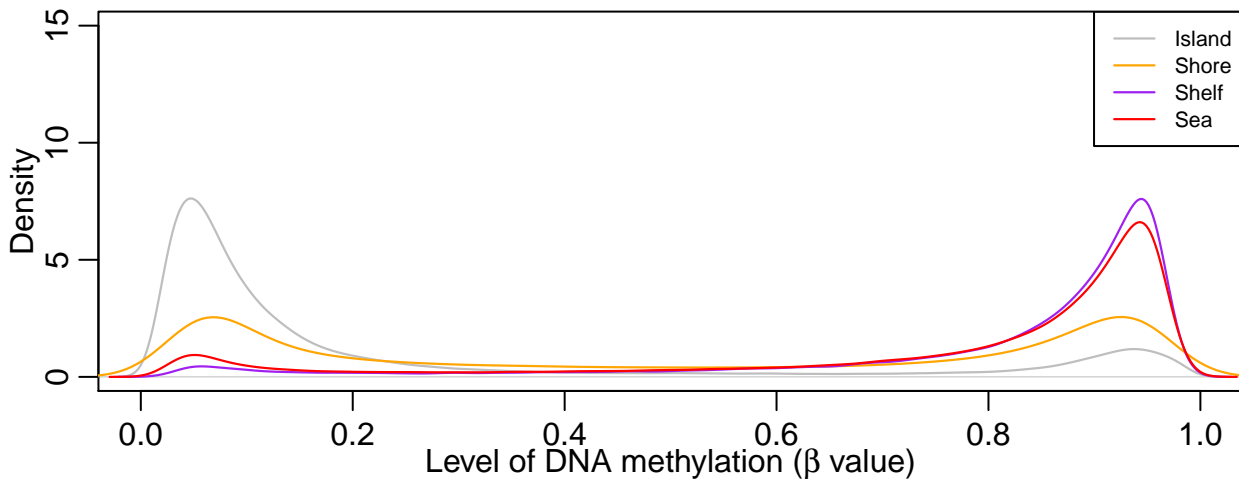

## HIL CpG classes

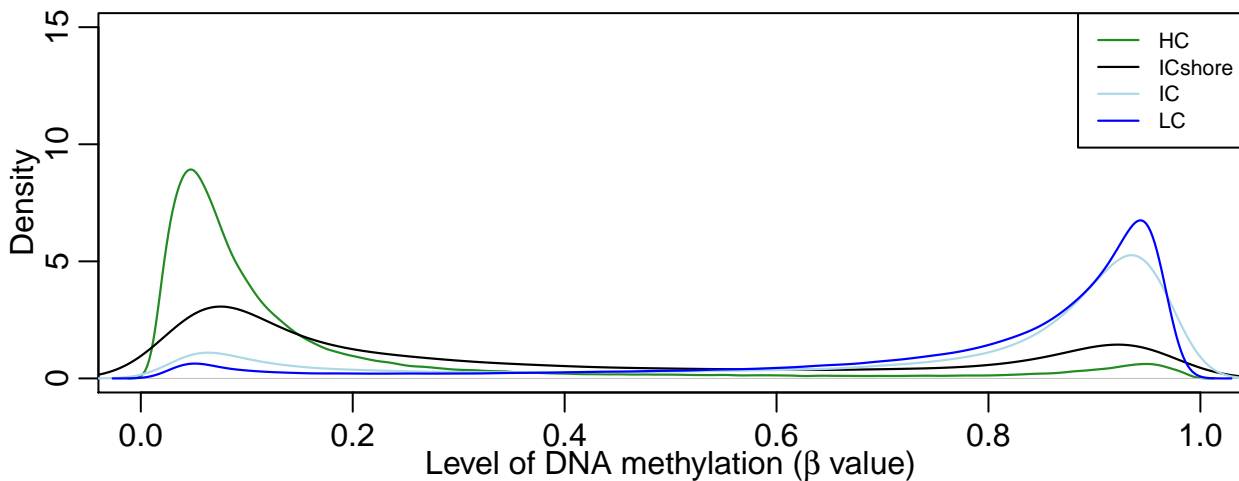

Supplement: Additional file 7 — Distinct patterns of DNAm across Illumina-annotated and HIL-annotated CpG classes in blood. Density curves were plotted using average ß values for probes within each Illumina-annotated and HIL-annotated CpG class in blood (n = 4). The number of probes contributing to each curve was: island = 136,712, shore = 100,083, shelf = 39,833, sea = 151,588, HC = 139,826, ICshore = 100,164, IC = 30,467 and LC = 157,759. For Illumina-annotated CpG classes, KS statistics in comparison to the distribution of DNAm of sea probes was 0.67 for island probes, 0.34 for shore probes and 0.06 for shelf probes. For HIL-annotated CpG classes, KS statistics in comparison to the distribution of DNAm of LC probes was 0.77 for HC probes, 0.53 for ICshore probes and 0.08 for IC probes. [file 1756-8935-6-4-S7.pdf]

# Distribution of DNA methylation in buccal

## Illumina CpG classes

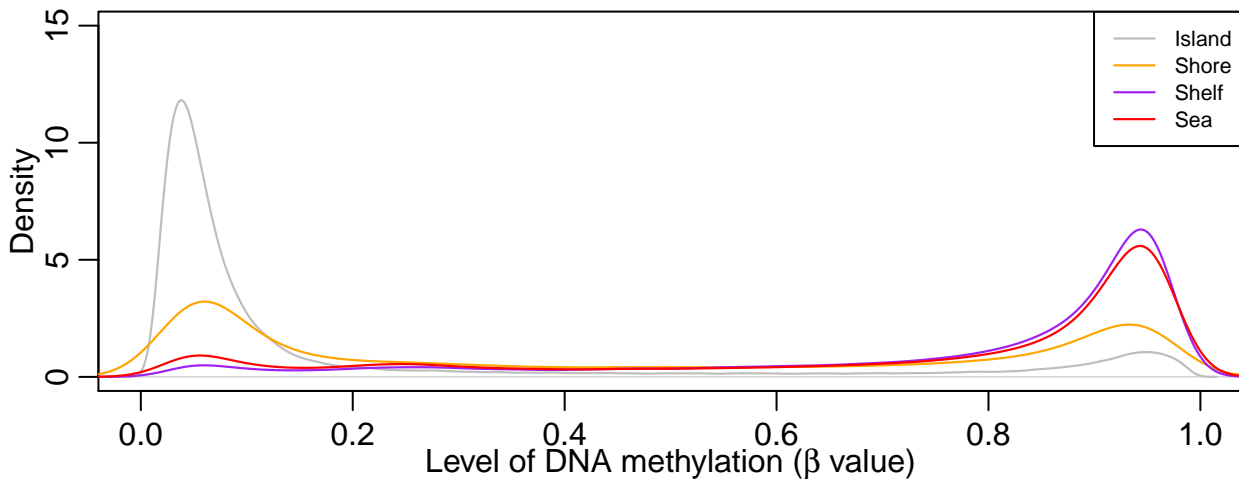

## HIL CpG classes

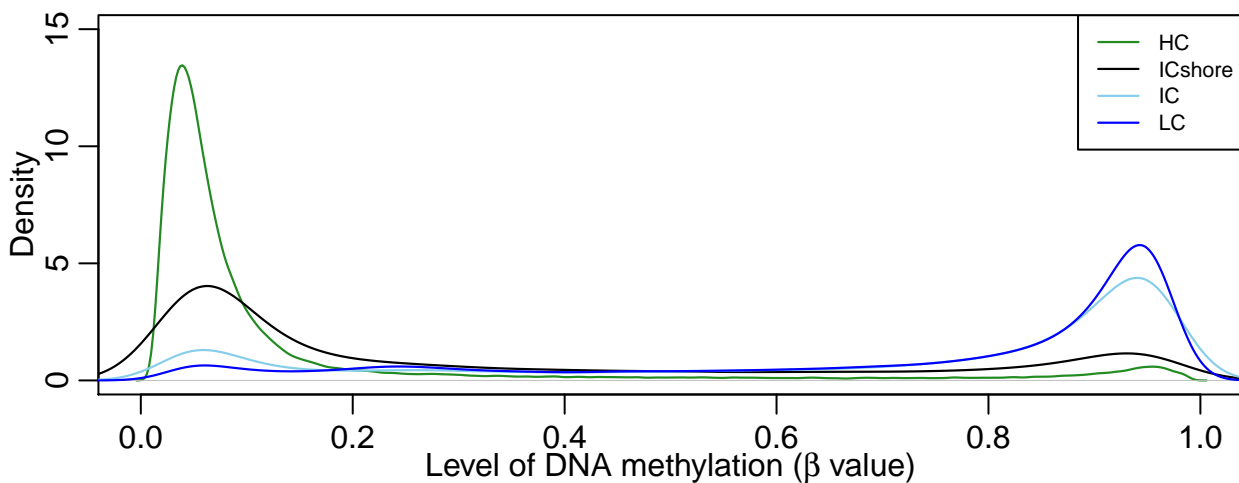

Supplement: Additional file 8 — Distinct patterns of DNAm across Illumina-annotated and HIL-annotated CpG classes in buccal samples. Density curves were plotted using average ß values for probes within each Illumina-annotated and HIL-annotated CpG class in buccal samples (n = 4). The number of probes contributing to each curve was: island = 136,712, shore = 100,083, shelf = 39,833, sea = 151,588, HC = 139,826, ICshore = 100,164, IC = 30,467 and LC = 157,759. For Illumina-annotated CpG classes, KS statistics in comparison to the distribution of DNAm of sea probes was 0.66 for island probes, 0.32 for shore probes and 0.06 for shelf probes. For HIL-annotated CpG classes, KS statistics in comparison to the distribution of DNAm of LC probes was 0.76 for HC probes, 0.49 for ICshore probes and 0.07 for IC probes. [file 1756-8935-6-4-S8.pdf]

# Distribution of DNA methylation in chorionic villi

## Illumina CpG classes

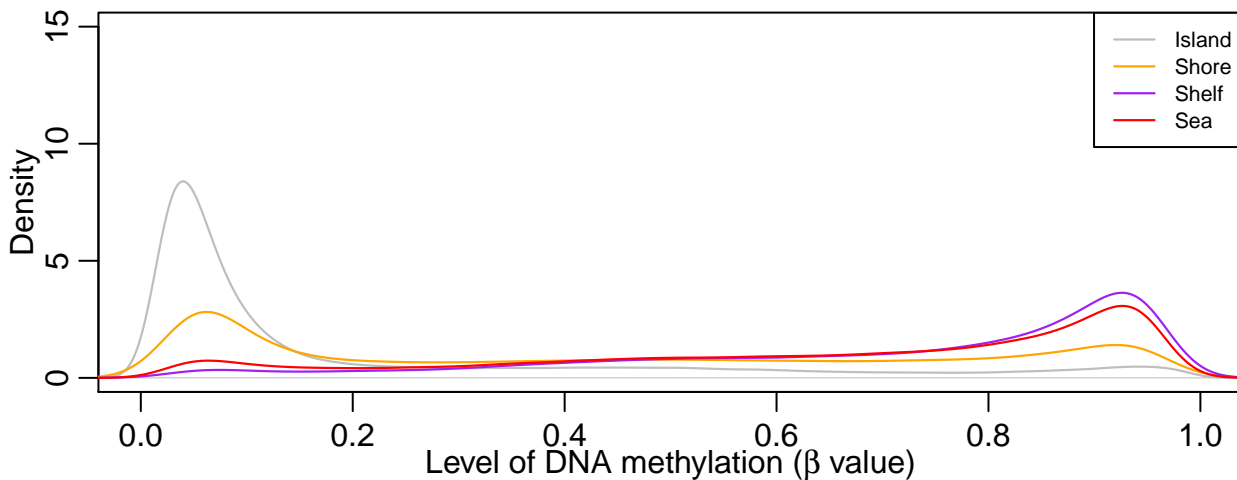

## HIL CpG classes

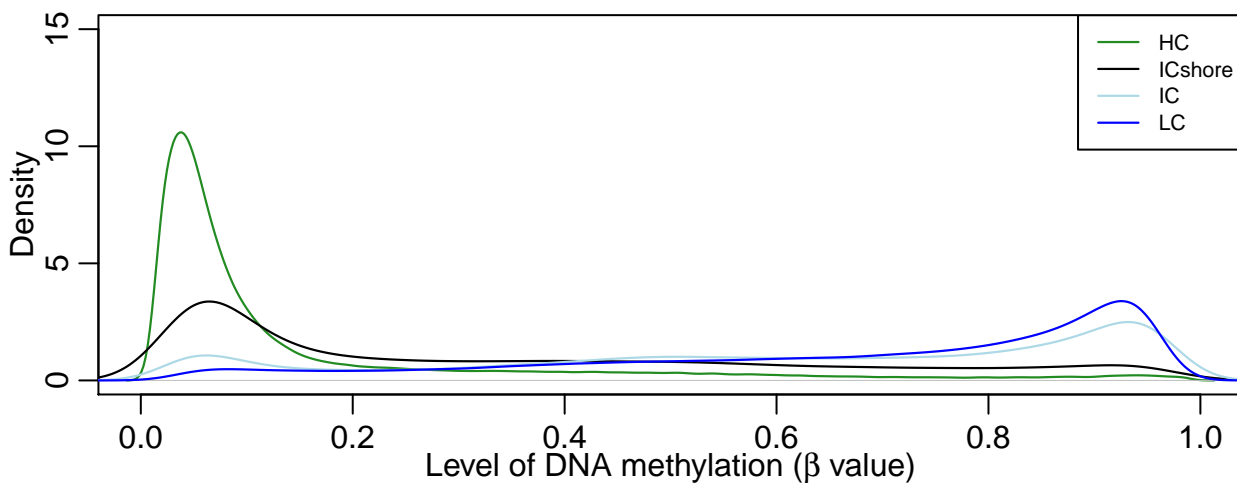

Supplement: Additional file 9 — Distinct patterns of DNAm across Illumina-annotated and HIL-annotated CpG classes in chorionic villi. Density curves were plotted using average ß values for probes within each Illumina-annotated and HIL-annotated CpG class in chorionic villi (n = 4). The number of probes contributing to each curve was: island = 136,712, shore = 100,083, shelf = 39,833, sea = 151,588, HC = 139,826, ICshore = 100,164, IC = 30,467 and LC = 157,759. For Illumina-annotated CpG classes, KS statistics in comparison to the distribution of DNAm of sea probes was 0.61 for island probes, 0.28 for shore probes and 0.08 for shelf probes. For HIL-annotated CpG classes, KS statistics in comparison to the distribution of DNAm of LC probes was 0.72 for HC probes, 0.45 for ICshore probes and 0.10 for IC probes. [file 1756-8935-6-4-S9.pdf]
